# Globe dislocation and optic nerve avulsion following all-terrain vehicle accidents

**DOI:** 10.1016/j.ajoc.2022.101621

**Published:** 2022-06-16

**Authors:** Amro Omari, Anaïs L. Carniciu, Maya Desai, Olivia Schimmel, Dianne M. Schlachter, Robert Folberg, Alon Kahana

**Affiliations:** a Oakland University William Beaumont School of Medicine, Royal Oak, MI, USA; b Beaumont Eye Institute, Royal Oak, MI, USA; c Consultants in Ophthalmic and Facial Plastic Surgery, Southfield, MI, USA; d Department of Pathology, Beaumont Health, Royal Oak, MI, USA; e Kahana Oculoplastic and Orbital Surgery, Ann Arbor, MI, USA

**Keywords:** All terrain vehicles, Globe dislocation, Optic nerve avulsion

## Abstract

**Purpose:**

Open-air motor vehicles present unique trauma risks to the eyes and face. We describe two patients who suffered a crash while riding an all-terrain vehicle (ATV), leading to globe dislocation with optic nerve avulsion in order to raise awareness about the risks associated with ATV accidents.

**Observations:**

In both cases, the injury was caused by high-speed trauma to the orbit involving a tree branch. One patient sustained a life threatening arrythmia requiring a short stay in the intensive care unit, and both patients required emergent surgical management and eventual socket reconstruction.

**Conclusions and Importance:**

These cases highlight the need for greater advocacy on behalf of rider safety. The authors encourage ophthalmologists to counsel patients who use ATVs to wear helmets, seatbelts, and protective eyewear to prevent these types of injuries in the future.

## 1 Introduction

Traumatic optic nerve avulsion is a rare clinical entity^1^ with a poor visual prognosis. Presentations in which avulsion should be suspected include globe subluxation or dislocation, decreased visual acuity or perception of light, and recent blunt force or penetrating trauma to the globe.^2^, ^3^, ^4^ Although most commonly described at the junction between the sclera and the globe,^5^ optic nerve avulsions can also occur more posteriorly at the orbital apex. Posterior avulsions can be associated with enucleation of the globe as well. Examples of published cases include reports of injuries from a dog bite, digital injury, or motor vehicle accidents.^2^^,^^3^ The common theme of such injuries is a penetrating trauma affecting the orbit posteriorly.

All-terrain vehicles (ATV) are open-air high-speed vehicles meant to be driven off road and with limited protection for the driver or passenger in case of an accident. These vehicles usually have no seatbelts or safety cages. Consequently, riders are at higher risk of high-speed trauma to the face from nearby structures as well as of being ejected from the vehicle. As such, these patients are at risk for optic nerve avulsions,^6^ a variety of injuries to the globe,^7^ and maxillofacial fractures.^8^ In addition, these patients can sustain life threatening complications,^9^ including arrythmias from the oculocardiac reflex.^10^ In the case of penetrating trauma associated with ejection from ATVs, the patient will likely need an orbital washout of vegetative matter^11^ or other foreign bodies to prevent potentially serious infections.

We describe two patients who sustained traumatic globe dislocation with optic nerve avulsion following an ATV crash and loss of the eye in each case. We hope to raise awareness of the risks to vision following these accidents. These case reports are compliant with HIPAA mandates and are in accordance with the tenets of the Declaration of Helsinki.

## 2 Findings

***Case 1****:* A 26-year-old woman was brought to our emergency room in a conscious state after an accident while driving an all-terrain vehicle (ATV). She was ejected (or thrown) from the vehicle and landed in a bush. She was not wearing a seatbelt, protective eyewear, or a helmet. She sustained both penetrating and blunt trauma to the left globe and orbit from tree branches.

**Fig. 1:**
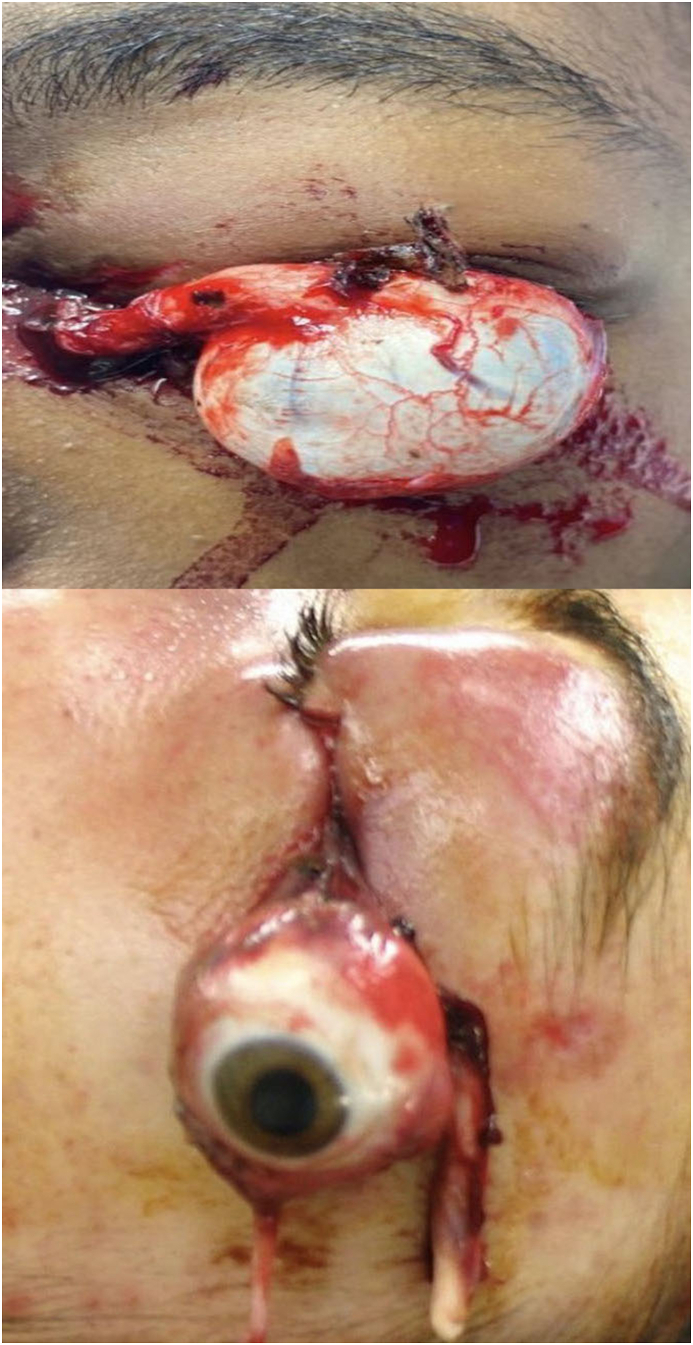
Clinical photographs of the two patients obtained before enucleation and orbital washout. Fig. 1a. Case 1: External photograph of the patient's left eye at the time of emergency room presentation. The globe is entirely dislocated out of the orbit and supported by the left cheek. The avulsed portion of the optic nerve has rotated anteriorly and is admixed with vegetative matter. Fig. 1b. Case 2: External photograph of patient's left eye upon presentation. The optic nerve is seen hanging at the superior edge of the enucleated globe. The lateral rectus muscle was still partially attached, with the globe rotated onto the cheek.

The left eye (OS) was displaced from the orbit with only a few extra ocular muscles still remaining attached (Fig. 1a). The patient could not perceive light from the left eye, had minimal extraocular motility, mild periorbital edema, and surrounding superficial eyelid lacerations. There was no evidence of retrobulbar hemorrhage or signs of infection. The optic nerve was severed with plant material adherent to the only part still remaining attached to the globe. She had an Ocular Trauma Score (OTS) of 1 (raw score 37).

The patient was taken to the operating room and underwent primary enucleation with exploration and washout of the orbit. During the procedure, the globe was found to remain attached to extra ocular muscles by tenon's capsule and conjunctiva. Vegetative foreign bodies (wood fragments and plant material) were found in the orbit. We therefore did not place an orbital implant in the socket, given the high risk of subsequent orbital infection. The patient was placed in the intensive care unit for several days after the surgery in order to monitor hemodynamic instability secondary to an oculocardiac reflex, for which she was managed with IV atropine. She was subsequently discharged four days following initial presentation and instructed to continue with outpatient management.

**Fig. 2:**
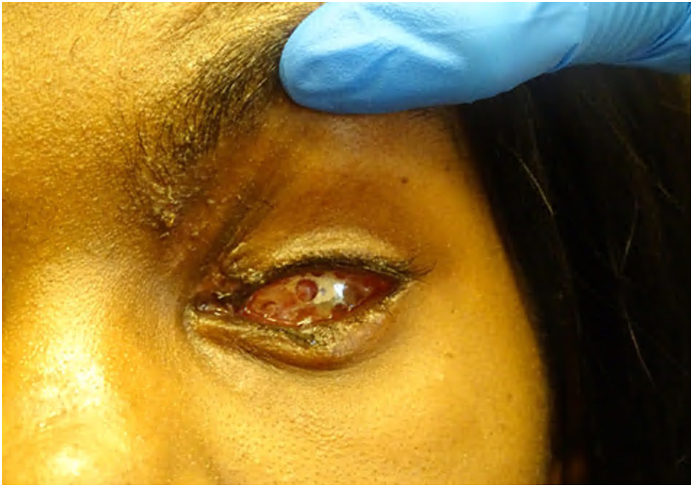
Clinical photograph obtained after the enucleation with washout but before reconstruction with the dermis fat graft. There is conjunctival dehiscence most pronounced temporally and inferior forniceal shortening. There are no signs of infection.

Two months following the initial injury, the patient underwent successful socket expansion with a dermis fat graft obtained from her abdomen. Fig. 2 depicts the pre op image before her reconstruction with the dermis fat graft. The patient is now a year and a half out from her initial injury and has a prosthetic in place. No other additional surgical interventions have taken place, as her socket has been stable.

**Fig. 3:**
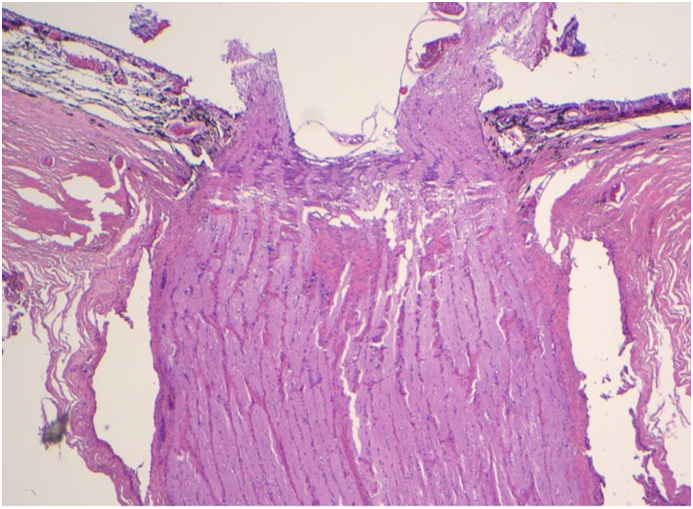

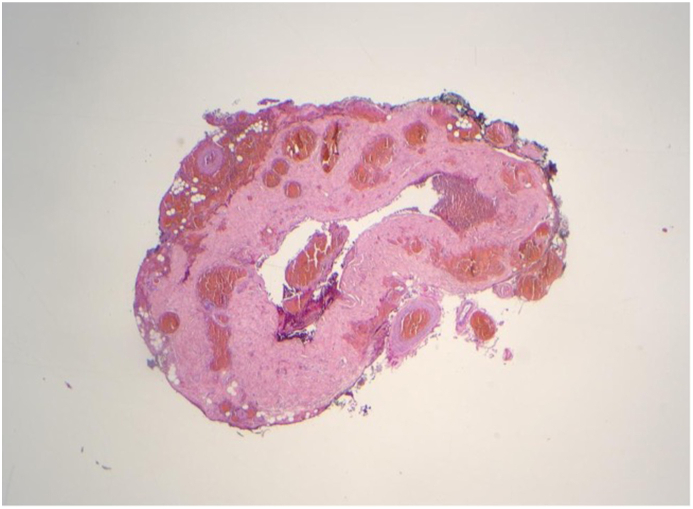
Histopathology specimens from Case 1 show that the optic nerve was avulsed rather than cut by the tree matter. Both specimens have been stained with Hematoxylin and Eosin. Fig. 3a is a 2x image illustrating the intraocular portion of the optic nerve. This portion was unremarkable and attached to its insertion at the optic nerve head. Fig. 3b is a 4x image of the transected portion of the optic nerve, which was located 16 mm behind its insertion in the optic nerve head. In this section, the dural sheath is intact with no signs of penetrating laceration but there is no remaining nerve tissue.

Microscopic examination of the enucleated eye revealed that the optic nerve was attached to the globe for a distance of 16 mm (Fig. 3a). At the distal end of the attached nerve, the dural sheath was collapsed but intact with no nerve tissue in the lumen (Fig. 3b). The absence of nerve tissue at this location provided evidence of an intraorbital disruption in the continuity of the optic nerve, an intraorbital avulsion.

***Case 2****:* A 16-year-old man was transferred to our care after an ATV accident with penetrating trauma to the left globe (Fig. 1b), classified with an OTS of 1 (raw score of 37). As in Case 1, the patient had not been wearing a helmet, protective eyewear, or a seatbelt at the time of injury. The patient's left eye was expulsed from the orbit. In the context of an optic nerve avulsion, the patient could not perceive light in that eye. Associated injuries included a margin-involving eyelid laceration, orbital floor fracture without signs of entrapment, and an arm fracture.

The patient was taken to the operating room to complete the enucleation and irrigate the traumatized orbit. The socket was repaired with a Medpor spherical implant. The floor fracture was repaired with a silicone sheet implant, and the eyelid lacerations were repaired. The patient sustained minimal blood loss and was stable from a systemic standpoint. Shortly after surgery, the patient was extubated and had an uncomplicated post-operative course. The patient was eventually fit with a custom prosthesis. He was followed for a total of 6 months after the initial injury, after which he decided to follow with a primary ophthalmologist closer to his home. The patient has also been stable with no additional surgical interventions.

## 3 Discussion

In both cases, exploration and washout of the traumatized orbits resulted in retrieval of vegetative matter. Fortunately, both had an unremarkable post-operative course without evidence of infection. Furthermore, both cases had a successful reconstruction of the anophthalmic socket with an acceptable cosmetic outcome.

## 4 Conclusion

We describe two cases of traumatic globe dislocation with optic nerve avulsion following an ATV crash in order to promote efforts to prevent these injuries. Both cases call attention to the serious risks associated with open-air high-speed motor vehicles such as ATVs. We encourage ophthalmologists to counsel patients who use ATVs to use adequate protective gear including full-faced helmets as well as eye goggles with a snug fit, foam cushioning to block out dust or foreign bodies, wide and durable lens material, and lenses with UV protection. Downhill ski helmets with goggles and motorcycle helmets with goggles represent good examples to emulate. Implementing these recommendations into official policies is likely to increase compliance with these vision and lifesaving interventions. Further enforcement may also be obtained with additional slogans or repetitive advertisements around ATV service institutions in order to remind people of the risks of not wearing protective gear when using these vehicles.

## Patient consent

Consent to publish the case report was not obtained. This report does not contain any personal information that could lead to the identification of the patient.

## Funding

The authors declare that the research and publication of their article was not funded financially by any supporting bodies or grants.

## Authorship

All authors attest that they meet the current ICMJE criteria for Authorship.

## Declaration of competing interest

The authors declare that there are no conflicts of interest, either financially or personally, regarding the publication of this paper.
